# Zoonotic tuberculosis in a high bovine tuberculosis burden area of Ethiopia

**DOI:** 10.3389/fpubh.2023.1204525

**Published:** 2023-09-13

**Authors:** Sosina Ayalew, Getinet Habtamu, Fantanesh Melese, Bamlak Tessema, Roland T. Ashford, Shubhada K. Chothe, Abraham Aseffa, James L. N. Wood, Stefan Berg, Adane Mihret, for the ETHICOBOTS Consortium, Abraham Aseffa

**Affiliations:** ^1^Armauer Hansen Research Institute, Addis Ababa, Ethiopia; ^2^Department of Biology, College of Natural Sciences, Arba Minch University, Arba Minch, Ethiopia; ^3^Department of Bacteriology, Animal and Plant Health Agency, Weybridge, United Kingdom; ^4^Department of Veterinary and Biomedical Sciences, The Pennsylvania State University, State College, PA, United States; ^5^Disease Dynamics Unit, Department of Veterinary Medicine, University of Cambridge, Cambridge, United Kingdom; ^6^Bernhard Nocht Institute for Tropical Medicine, Hamburg, Germany

**Keywords:** *Mycobacterium bovis*, *Mycobacterium tuberculosis*, zoonosis, cattle, central Ethiopia

## Abstract

**Background:**

Tuberculosis (TB) is a major cause of ill health and one of the leading causes of death worldwide, caused by species of the *Mycobacterium tuberculosis* complex (MTBC), with *Mycobacterium tuberculosis* being the dominant pathogen in humans and *Mycobacterium bovis* in cattle. Zoonotic transmission of TB (zTB) to humans is frequent particularly where TB prevalence is high in cattle. In this study, we explored the prevalence of zTB in central Ethiopia, an area highly affected by bovine TB (bTB) in cattle.

**Method:**

A convenient sample of 385 patients with pulmonary tuberculosis (PTB, *N* = 287) and tuberculous lymphadenitis (TBLN, *N* = 98) were included in this cross-sectional study in central Ethiopia. Sputum and fine needle aspirate (FNA) samples were obtained from patients with PTB and TBLN, respectively, and cultures were performed using BACTEC^™^ MGIT^™^ 960. All culture positive samples were subjected to quantitative PCR (qPCR) assays, targeting IS*1081*, RD9 and RD4 genomic regions for detection of MTBC, *M. tuberculosis* and *M. bovis*, respectively.

**Results:**

Two hundred and fifty-five out of 385 sampled patients were culture positive and all were isolates identified as MTBC by being positive for the IS*1081* assay. Among them, 249 (97.6%) samples had also a positive RD9 result (intact RD9 locus) and were consequently classified as *M. tuberculosis*. The remaining six (2.4%) isolates were RD4 deficient and thereby classified as *M. bovis*. Five out of these six *M. bovis* strains originated from PTB patients whereas one was isolated from a TBLN patient. Occupational risk and the widespread consumption of raw animal products were identified as potential sources of *M. bovis* infection in humans, and the isolation of *M. bovis* from PTB patients suggests the possibility of human-to-human transmission, particularly in patients with no known contact history with animals.

**Conclusion:**

The detected proportion of culture positive cases of 2.4% being *M. bovis* from this region was higher zTB rate than previously reported for the general population of Ethiopia. Patients with *M. bovis* infection are more likely to get less efficient TB treatment because *M. bovis* is inherently resistant to pyrazinamide. MTBC species identification should be performed where *M. bovis* is common in cattle, especially in patients who have a history of recurrence or treatment failure.

## 1. Introduction

Tuberculosis (TB) is among the most significant human infectious diseases worldwide, especially impacting low- and middle-income countries. An estimated 10.6 million new cases and 1.6 million deaths were attributed to TB in 2021 (1). Although the vast majority of TB cases in humans are caused by *Mycobacterium tuberculosis sensu stricto*, other highly related subspecies of the *Mycobacterium tuberculosis* complex (MTBC), such as *Mycobacterium africanum* and *Mycobacterium bovis* can also cause TB in humans. In fact, all species within the MTBC share over 99.9% identity at the genome level (2). Despite this high similarity however, there appears to be host-adaptation among the different MTBC species (3), with *M. bovis* being mainly associated with TB in cattle, also known as bovine TB (bTB).

It has been estimated that 1.4% of all human TB cases in the world, and 2.8% of all cases in the African population, are attributed to *M. bovis* (4, 5). However, the global picture of human TB caused by *M. bovis* is largely incomplete because of reliance on laboratory techniques that are insufficient for accurate differentiation between *M. bovis* and *M. tuberculosis*, including direct smear microscopy, GeneXpert, or culturing of mycobacteria without species-level identification (6, 7). Moreover, human TB caused by *M. bovis* is clinically, radiographically, and pathologically indistinguishable from TB caused by *M. tuberculosis* (8, 9). Hence, the exact contribution of *M. bovis* to the global epidemiology of human TB is possibly underestimated because of underdiagnosis and underreporting, particularly in developing countries where bTB is endemic in cattle and likely not controlled for.

Zoonotic tuberculosis (zTB) has previously been defined as human infection with *M. bovis* (4). More recently, other subspecies of the MTBC have also been identified in cattle and the definition of zTB has been challenged (10). However, for the purpose of this Ethiopian study, we refer to zTB as “TB in humans caused by *M. bovis*” and bTB as “TB in cattle caused by *M. bovis*”. Transmission of zTB to humans occurs most frequently through inhalation of aerosol droplets from infected animals, or through ingestion of untreated dairy products carrying *M. bovis* (7). Human-to-human transmission is considered significantly less common (11, 12).

Ethiopia is a highly agrarian society, with over 70% of its nearly 120 million people engaged in agriculture. With an estimated 65 million cattle, its livestock sector has the largest national cattle herd in Africa and the sixth largest in the world (13). Approximately 98% of these cattle are of the local zebu breeds reared extensively by rural smallholders or pastoralists, while the remaining 2% are dairy cattle of exotic breeds—or crosses with the local zebus—that are mostly accommodated in intensive husbandry settings around urban centers (13). Extensive epidemiological studies from the last decades suggest that the prevalence of bTB in cattle in rural settings across Ethiopia is relatively low, with rates of 5–10% (14, 15). However, the intensive dairy sector has been more affected. Several studies in the well-established dairy belt around Addis Ababa in central Ethiopia have reported an average bTB animal prevalence of between 25 and 30%, while the herd prevalence in certain parts has reached 50–60% bTB (16–18). Ethiopia has not yet implemented a bTB intervention program. Only sporadic tuberculin testing and subsequent slaughter of infected cattle have been performed in selected herds but at small scale. Also, a basic post-mortem examination program at slaughterhouses has been introduced. Previous attempts to estimate the prevalence of *M. bovis* in the human population in Ethiopia [which has a TB incidence rate of 143/100,000 population (1)] have suggested a zTB rate of below 1% (19, 20). Most of the sites explored in these studies have however been in regions of the country where the level of bTB in cattle has been relatively low, while studies focusing on zTB in the central parts of the country, where the bTB rate is very high, have been limited. Therefore, we set out to investigate the prevalence of zTB in the central region of Ethiopia to understand whether the very high bTB prevalence in cattle is reflected in the human population. We also discuss the zoonotic impact from the perspective of exposure to cattle and the behavior of raw milk and meat consumption.

## 2. Materials and methods

### 2.1. Study design and setting

**Figure 1:**
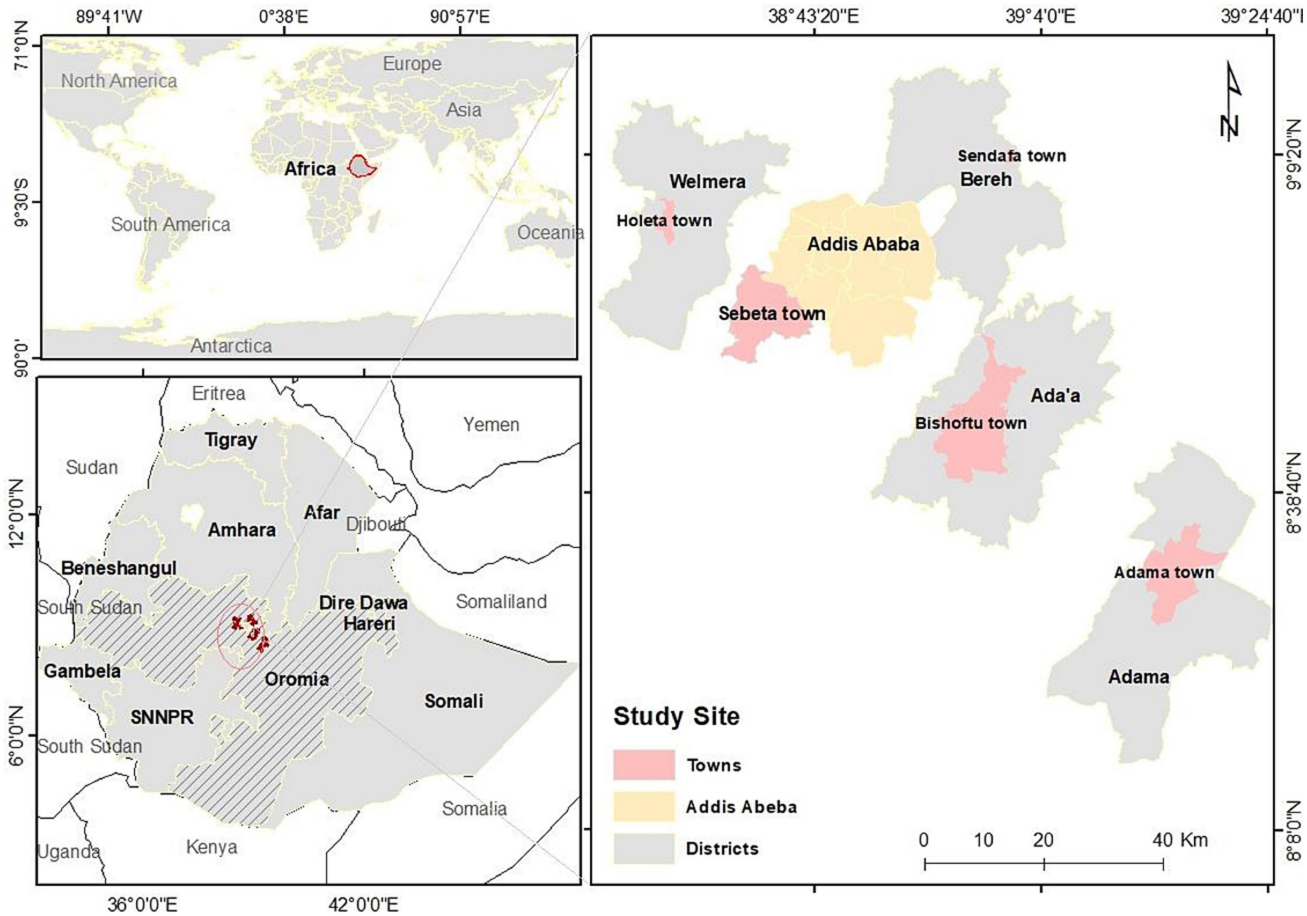
Mapping of seven health centers in Addis Ababa and five surrounding towns in central Ethiopia where patients were recruited into this study.

A multi-center health facility-based cross-sectional study was conducted in central Ethiopia from October 2019 to March 2021. Three hospitals (Adama, ALERT and Bishoftu) and five health centers (Adama, Bishoftu, Sebeta, Holeta, and Sendafa) located in Addis Ababa city and the surrounding zone of Oromiya region were selected to recruit study participants. The high prevalence of bTB in cattle in central Ethiopia was the major reason for this selection. Health centers typically serve 50,000–60,000 people, whereas Bishoftu and Adama hospitals each serve approximately 1.2–1.5 million. An illustrative representation of the study areas is depicted in Figure 1.

### 2.2. Study population

Patients clinically or microbiologically diagnosed with pulmonary TB (PTB) or TB lymphadenitis (TBLN) were enrolled consecutively upon informed consent. PTB cases were enrolled at all selected study sites whereas TBLN patients were enrolled in hospitals where fine-needle aspirate (FNA) cytology examination by a pathologist was available. Extrapulmonary TB (EPTB) patients other than those with TBLN were excluded from the study.

### 2.3. Data collection and sampling of clinical specimens

Clinical and demographic information was collected from enrolled patients using a structured questionnaire. Morning sputum and FNA specimens were collected before the initiation of anti-TB treatment from PTB and TBLN patients, respectively. FNA specimens were collected aseptically by experienced pathologists from enlarged cervical lymph nodes with a 21-gage needle attached to a 10 mL syringe and transferred into cryo-tubes containing 1 mL phosphate buffer saline (PBS) pH 7.2, while sputum specimens were collected in sterile 50 mL plastic tubes. Both sample types were kept at −20°C at the study sites until they were transported on ice (up to +4°C) to the Armauer Hansen Research Institute (AHRI) in Addis Ababa for sample processing and culturing of mycobacteria.

### 2.4. Culturing of mycobacteria

**Figure 2:**
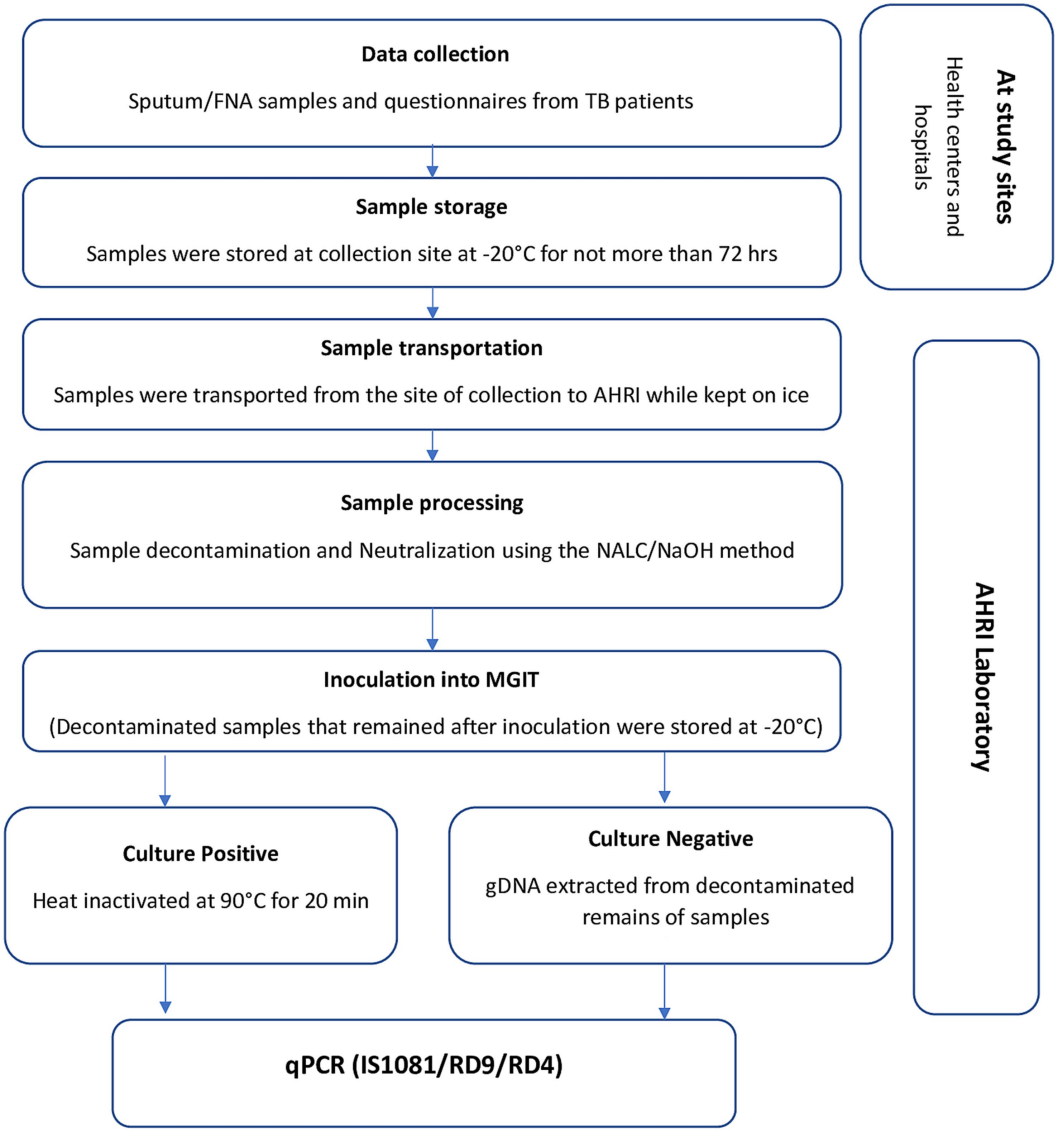
Overview of data collection and the laboratory methods.

Mycobacterial culturing was performed on sputum and FNA samples following the procedure indicated in the Mycobacteriology Laboratory Manual (21) using BACTEC^™^ MGIT^™^ 960 Mycobacterial detection system. In brief, samples were decontaminated by the standard N-acetyl-L-cysteine and sodium hydroxide (NALC/NaOH) method with a final NaOH concentration of 1%. An equal volume of standard NALC/NaOH solution was added to the specimen and incubated for 15 min. After neutralization with PBS and 15 min centrifugation at 3,000 × g, the sediment was re-suspended in 1 mL of sterile PBS. A volume of 0.5 mL of each re-suspended sample was inoculated into a MGIT liquid medium tube. Inoculated MGIT tubes were placed directly into the MGIT 960 instrument for incubation for up to 48 days or until detection of growth. Heat-killed cells were prepared, by taking 500 μL broth from culture-positive MGIT tubes for incubation at 90°C for 20 min, and used for subsequent molecular identification. In parallel, decontaminated sample volumes remaining after MGIT inoculation were used for Ziehl-Neelsen staining and smear microscopy, and for culture-negative samples, if enough volume remained, genomic DNA (gDNA) was extracted using QIAamp^®^ DNA mini kit (Qiagen) following the manufacturer’s protocol and eluted in a volume of 50 μL, and then stored at −20°C until further analysis. A flow chart of the sample collection, processing, and molecular typing is provided in Figure 2.

### 2.5. Identification of mycobacterial genomic DNA

Quantitative PCR (qPCR) was performed on heat-killed bacterial suspensions or extracted gDNA in the case of culture-negative samples. IS*1081*, RD9, and RD4 were used as target genomic regions for identification of MTBC, *M. tuberculosis*, and *M. bovis*, respectively. IS*1081* is an insertion sequence specific for all mycobacterial species of the MTBC and has been shown to appear as six copies in each genome (22). The IS*1081* assay is expected to be more sensitive than the RD9 and RD4 assays since it is a multiple copy gene (22). Therefore, the qPCR assay with specific primers for IS*1081* was used as a screening test to identify the presence of genomes from the MTBC in the samples. Samples that tested positive for IS*1081* were then tested by qPCR for the presence of RD9 and RD4, for further species identification. The assay for IS*1081* was based on a protocol published by Dykema et al. (23) and the assays for RD9 and RD4 were based on the protocols described by Halse et al. (24) and King et al. (25), respectively.

The reaction mixture for the IS*1081* assay was 10 μL PrimeTime Gene Expression Master Mix (2X) (Integrated DNA Technologies, Inc), 0.5 μL PrimeTime qPCR Assay (40X) consisting of premixed primers and probe of IS*1081*_F 5′-GGC TGC TCT CGA CGT TCA TC-3′; IS*1081_R* 5′-CGC TGA TTG GAC CGC TCA T-3′, IS*1081_P* [6FAM] CTG AAG CCG ACG CCC TGT GC [BHQ1], 4.5 μL nuclease free water, and 5 μL template DNA in a final volume of 20 μL. Reaction mixture for the RD4 assay was 2.5 μL of 10 μM RD4_FW 5′- TGT GAA TTC ATA CAA GCC GTA GTC G -3′, 2.5 μL of 10 μM RD4_Rev 5′- CCC GTA GCG TTA CTG AGA AAT TGC -3′, 0.5 μL 10 μM RD4_Probe [6FAM]-AGC GCA ACA CTC TTG GAG TGG CCT AC-[BHQ1], 12.5 μL of TaqMan^®^ Environmental Master Mix 2.0 (Applied Biosystems, [Thermo fisher]), 2 μL nuclease free water and 5 μL template DNA in a final volume of 25 μL. A similar reaction mixture was used for the RD9 assay with primer/probe sequence as follows: RD9_FW 5′-TGC GGG CGG ACA ACT C-3′, RD9_Rev 5′-CAC TGC GGT CGG CAT TG-3′, RD9_Probe [Cy5]-AGG TTT CAC CTT CGA CCC-[BHQ2]. The PCR cycling conditions for the IS*1081* assay were 3 min at 95°C for enzyme activation, followed by 15 s at 95°C for denaturation and 1 min at 63°C for annealing and extension involving a total of 40 cycles. RD4/RD9 assays were performed at 50°C for 2 min, followed by 95°C for 10 min and then 40 cycles of 95°C for 15 s and 58°C for 1 min at which the fluorescence acquisition was performed. All samples were tested in duplicate, and average IS1081 Ct values less than 37 were considered positive. The reactions were performed using a Rotor-Gene (RG-3000).

### 2.6. Quality control

Standard operational procedures for all laboratory tests were employed uniformly throughout the study. To prevent possible contamination of qPCR assays, sample preparation and DNA extraction, qPCR master mix preparation, and qPCR amplification were carried out in three separate rooms using dedicated laboratory coats, pipettes and sterile tips. Furthermore, purified gDNA of *M. tuberculosis* H_37_Rv and *M. bovis* BCG and sterile molecular grade water were used as positive and negative controls in each qPCR round.

### 2.7. Data entry and analysis

All demographic and laboratory data collected were entered into a Microsoft Excel spreadsheet and verified. The questionnaire and laboratory data were linked by a unique identification code. SPSS statistical software version 27 (IBM Corp., Armonk, N.Y., United States) was used for analysis. Frequencies and cross-tabulation were used to summarize descriptive statistics. Bivariate and multivariable logistic regression analysis were applied to determine the significance among categorical variables. A *p-*value less than 0.05 was considered statistically significant.

### 2.8. Ethical considerations

The study was approved by AHRI/ALERT Ethics Review Committee (AAERC) (Ref. No: 301/001/2015). Study participants were provided with adequate information about the project and its commitments before signing informed consent.

## 3. Results

### 3.1. Characteristics of the study population

A total of 385 participants were enrolled in this cross-sectional study, including 287 PTB and 98 TBLN patients, stratified across six study sites as listed in Table 1 and mapped in Figure 1. The demographic analysis (Table 2) indicated that PTB was more frequent in males, while TBLN was more frequent in females (*p* ≤ 0.01), with male-to-female ratios of 1.9:1 and 0.8:1, respectively. The mean age of all participants was 33 ± 14 years, while 270 (70%) of the study participants were in the age group between 20 and 45 years. With regards to consumption behavior of raw milk and raw meat among all interviewed patients, 64% of the respondents said that they consumed raw milk whereas 77% consumed raw meat; in total 52% of them consumed both raw milk and raw meat. There was no notable difference in milk consumption between patients of the two disease types, however, eating raw meat was significantly less common among the TBLN patients (OR = 0.4, CI 95% 0.2–0.7; *p* < 0.01). TBLN cases were more frequent among study participants who reported close contact with animals (OR = 2.3, CI 95% 1.3–3.9; *p* < 0.001) as compared to those who did not.

**Table 1:** Number of enrolled pulmonary TB and TB lymphadenitis patients with clinical TB symptoms stratified by collection site and rates of culture-positivity.

Collection site	Pulmonary TB		TB Lymphadenitis	
	No of patients	Culture-positive	No of patients	Culture-positive
Adama	75	53 (70.7%)	32	14 (43.8%)
Bishoftu	166	116 (69.9%)	45	31 (68.9)
Sendafa	19	13 (68.4%)	0	0
Holeta	10	10 (100%)	0	0
Sebeta	17	6 (35.3%)	0	0
Addis Ababa	0	0	21	12 (57.1%)
Total	287	198 (69.0%)	98	57 (58.2%)

**Table 2:** Demographic and clinical characteristics of the study participants from central Ethiopia (*n* = 385).

Patient characteristics		PTB N (%)	TBLN N (%)	Crude OR 95%CI	Adjusted OR 95%CI
Sex^a^	Male	188 (65.5%)	43 (43.9%)		
	Female	99 (34.5%)	55 (56.1%)	2.4 (1.5–3.9)^***^	2.0 (1.2–3.5)^**^
Age^b^	<20	27 (9.5%)	21 (21.4%)		
	20–45	207 (72.6%)	63 (64.3%)	0.4 (0.2–0.7)^**^	0.4 (0.2–0.9)^*^
	>45	51 (17.9%)	14 (14.3%)	0.4 (0.2–0.8)^*^	0.5 (0.2–1.1)
Previous TB history	No	237 (82.6%)	78 (79.6)		
	Yes	40 (13.9%)	14 (14.3%)	1.1 (0.6–2.1)	1.1 (0.6–2.4)
	Unknown	10 (3.5%)	6 (6.1%)	1.8 (0.6–5.2)	4.3 (1.1–16.2)^*^
History of BCG vaccination	No	177 (61.7%)	70 (71.4%)		
	Yes	54 (18.8%)	23 (23.5%)	1.1 (0.6–1.9)	1.3 (0.7–2.5)
	Unknown	56 (19.5%)	5 (5.1%)	0.2 (0.1–0.6)^**^	0.2 (0.1–0.5)^***^
Raw Milk consumption	No	100 (34.8%)	25 (25.5%)		
	Yes	175 (61.0%)	72 (73.5%)	1.6 (0.9–2.8)	1.6 (0.9–3.2)
	Unknown	12 (4.2%)	1 (1.0%)	0.3 (0.0–2.7)	0.5 (0.1–4.1)
Raw meat consumption	No	49 (17.1%)	34 (34.7%)		
	Yes	236 (82.2%)	62 (63.3%)	0.4 (0.2–0.6)^***^	0.4 (0.2–0.7)^**^
	Unknown	2 (0.7%)	2 (2.0%)	1.4 (0.2–10.7)	1.4 (0.2–14.1)
Level of contact with cattle	Not close	122 (42.5%)	24 (24.7%)		
	Moderate	33 (11.5%)	14 (14.4%)	2.2 (1.0–4.6)^*^	1.4 (0.6–3.4)
	Very close	132 (46.0%)	59 (60.8%)	2.3 (1.3–3.9)^**^	2.2 (1.2–4.1)^*^

a Adjusted for age and site alone.

b Adjusted for sex and site alone.

*p ≤ 0.05; **p ≤ 0.01; ***p ≤ 0.001.

### 3.2. Culturing and typing of *Mycobacterium tuberculosis* complex

Culturing of mycobacteria from sputum and FNA samples using the MGIT system yielded 198 (68.9%) and 57 (58.2%) isolates, respectively. All culture positive samples were also positive for acid-fast bacilli as shown by ZN staining. Molecular typing by qPCR was performed on all 255 culture positive samples and all were first identified as MTBC by being positive for the IS*1081* assay. Among them, 249 (97.6%) samples had also a positive RD9 result (intact RD9 locus) and were subsequently classified as *M. tuberculosis*, while the remaining six culture positive MGIT samples (2.4%) were identified as both RD9 and RD4 deficient and thereby classified as *M. bovis*. Five out of these six *M. bovis* isolates originated from PTB patients whereas one was sampled from a TBLN patient (Table 3, Supplementary Table S1). All six patients identified with *M. bovis* infection in this study were males among whom four had occupations associated with animal handling (Table 4).

**Table 3:** Molecular identification of disease agents by qPCR in culture-positive and culture-negative samples stratified by type of TB disease.

Samples processed for culture	Pulmonary TB (Sputum smear −) *N* = 180	Pulmonary TB (Sputum smear +) *N* = 107	TB lymphadenitis (FNA) *N* = 98	Total *N* = 385
Culture positive	93 (51.7%)	105 (98.1%)	57 (58.2%)	255 (66.2%)
Molecular typing	93	105	57	255
*M. tuberculosis*	90 (96.8%)	103 (98.1%)	56 (98.2%)	249 (97.6%)
*M. bovis*	3 (3.2%)	2 (1.9%)	1 (1.8%)	6 (2.4%)
Negative	0 (0.0%)	0 (0.0%)	0 (0.0%)	0 (0.0%)
Culture negative	87 (48.3%)	2 (1.9%)	41 (41.8%)	130 (33.8%)
Molecular typing*	78	2	35	115
*M. tuberculosis*	24 (30.8%)	1 (50%)	5 (14.3%)	30 (26.1%)
*M. bovis*	0	0	0	0
MTBC	39 (50%)	1 (50%)	8 (22.9%)	48 (41.7%)
Negative	15 (19.2%)	0	22 (62.8%)	37 (32.2%)
Identification rate	86.7%	100%	71.4%	86.5%

MTBC, Mycobacterium tuberculosis complex species; FNA, fine-needle aspirate.

*Typing of a culture-negative sample was dependent on access to gDNA from the original processed sample.

**Table 4:** Sociodemographic and clinical characteristics of the six study participants identified with *Mycobacterium bovis* infection.

Patient characteristics	Patient 1	Patient 2	Patient 3	Patient 4	Patient 5	Patient 6
Gender	Male	Male	Male	Male	Male	Male
Age (yr)	13	38	23	38	46	34
Occupation	Student	Employee	Veterinarian	Animal attendant	Farmer	Meat seller
Location	Addis Ababa	Bishoftu	Sebeta	Sebeta	Bishoftu	Bishoftu
Previous TB history	No	No	No	No	No	No
Raw milk consumption	No	No	No	No	Yes	Yes
Raw meat consumption	No	No	No	Yes	Yes	Yes
Level of cattle contact	Not close	Not close	Very close	Very close	Very close	Very close
Type of TB disease^*^	TBLN	PTB+	PTB−	PTB−	PTB−	PTB+

*PTB+, Pulmonary TB smear+; PTB−, Pulmonary TB smear−; TBLN, TB Lymphadenitis.

In attempts to identify the causative agents among TB patients with culture-negative results, gDNA was extracted from the remains of their sputum and FNA samples for 115 out of 130 patients and the extracted gDNA was used for molecular typing by qPCR. Thirty of these 115 samples (26.1%) were typed as *M. tuberculosis* as shown by IS*1081* being present and RD9 intact. Samples confirmed as positive for the IS*1081* assay, but with a negative result for the RD9 and RD4 assays, were classified as MTBC with no further characterization and accounted for 48/115 (41.7%) of the culture-negative cases. All of these samples had a Ct value between 31 and 37 for IS*1081* qPCR. The remaining 37 culture negative samples were negative also for IS*1081* by the qPCR assay (Supplementary Table S2). None of the patients with culture-negative results but with IS*1081* positive results was identified as *M. bovis*, as determined by gDNA extraction and qPCR.

Considering all samples that were identified as MTBC by culture and subsequent qPCR typing, or by direct qPCR typing, the overall identification rate in this study was 86.5% with 100% identification for smear-positive PTB, 86.7% for smear-negative PTB, and 71.4% for TBLN (Table 3).

## 4. Discussion

This study was designed to better understand the prevalence of zTB in humans living in central Ethiopia where bTB is highly prevalent in cattle. bTB has likely been endemic in Ethiopian cattle since records began nearly 50 years ago (26), and a high rate of bTB in the intensive dairy sector in central Ethiopia has been documented over at least the last 15 years (16–18), suggesting that the human population in this area has been highly exposed to bTB for a long time and that the risk of zTB is significant. In the present study, the overall *M. bovis* prevalence among 255 culture-positive cases was 2.4%, which is higher than previous reports from Ethiopia that have only reported a handful of *M. bovis* cases among much larger study populations, leading to estimated zTB rates far below 1% (19, 20). Interestingly though, these latter studies have, to a large extent, only explored human populations living in areas where extensive cattle husbandry is dominant, with mainly cattle of zebu breeds. As tuberculin testing and abattoir surveys of zebu cattle in these areas of Ethiopia have mostly reported relatively low bTB rates of 0–5% (14, 15) and rarely above 10% (15, 27), it is tempting to suggest that these lower rates in cattle lead to a lower risk of zTB transmission from the zebus which could explain the overall low zTB rates of <1% in Ethiopia (20). Local zebus appear to have higher resistance to bTB than exotic cattle breeds (28, 29). The introduction of exotic breeds and high-yield dairy systems into African nations was not without controversy because it may not have adequately taken ecological and cultural variations into account. In Africa, the dietary habit of the people, close physical contact between animals and humans, and inadequate bTB control in animals have facilitated transmission of the disease between animals and humans (30). In Ethiopia, the national herd of exotic Holstein-Friesian cattle has increased over many decades driven by the country’s significant need for increased milk production in urban areas. However, higher risk of zTB has been advised, as a consequence thereof, due to intensive rearing of these exotic breeds that are likely more susceptible to bTB (29, 31). Approximately ten times higher bTB rates in cattle recorded in the dairy belt in central Ethiopia, which is dominated by exotic breeds, may correlate well with the significantly higher zTB prevalence in the human population (2.4%) living in that area, as we are reporting here. In fact, five of the six human cases in this study lived in Sebeta and Bishoftu, two sites that have recorded >70% bTB prevalence at herd level (17). Due to free cattle movement in Ethiopia and lack of a bTB control program, such as a test-and-movement regulation to avoid bTB infected cattle being dispersed, it is inevitable that bTB will spread from the central region to other regions with lower prevalence. This is in particular concerning the expansion of the intensive dairy sector to new urban centers across the country. A further shift to intensive dairy production with more exotic or cross-bred cattle, without any interventions, will likely increase the risk for zTB in the Ethiopian population.

Earlier publications on zTB in Ethiopia (19, 32) have tried to explain the extremely high rate of EPTB reported in Ethiopia (with regional variation between 20 and 45% and dominated by TBLN) by the national cattle herd being endemic for bTB and by subsequent transmission to humans through common raw milk and meat consumption. However, Berg et al. (32) concluded that bTB may contribute to the high EPTB rates, but that it is not the main factor. Their initial hypothesis that high endemic bTB rates in cattle would be reflected in the human population was largely based on historical figures on bTB. Epidemiological work during the first half of the 20th century showed that the agrarian societies in Europe suffered heavily from bTB in their national cattle herds, with average animal rates between 20 and 40% commonly reported (33), especially among the intensively reared dairy herds. In parallel, many European countries saw human TB incidence rates above 200/100,000 population (similar to those recorded in Ethiopia over the last few decades). Based on the methodologies available at that time for distinguishing between the two TB pathogens—*M. tuberculosis* and *M. bovis*—it was estimated that approximately 10–15% of TB in humans was caused by the bovine version of the TB bacilli (at that time not yet named *M. bovis*) (33, 34) through consumption of unpasteurised dairy products or interaction with infected cattle herds. This assessment was reinforced by the identification of the bTB version in humans to a higher degree among EPTB cases than among PTB cases (35, 36), suggesting transmission from cattle by ingestion of infected milk rather than aerosol transmission through inhalation of the bacilli. Despite similar circumstances that could lead to high zTB transmission, there might be several explanations why we cannot translate these epidemiological figures from Europe a century ago to the current situation in Ethiopia. One argument is that the ability to correctly identify the disease agent has improved. In Europe at that time, species identifications were mainly based on phenotypic characteristics of the disease agents, such as differentiation by colony morphology and biochemical tests (37). These methods were less accurate and reproducible as compared to the current genotyping methods that have been developed after the genomic characterisations of all known species within the MTBC (2, 38). Therefore, the rates of zTB reported from Europe about a century ago may be less reliable, thereby making a comparison with current genotyping difficult. Since the present study used a combination of improved culturing yield through the MGIT system and a highly sensitive genotyping technique, the results that we report here are more likely to be closer to the true prevalence of *M. bovis* in humans in central Ethiopia.

Another question relates to the disease exposure of zTB among Ethiopians. A large proportion of our study population had a habit of consuming untreated milk (64%) and raw meat (77%). In many Ethiopian communities, untreated (raw) milk is consumed by many, mainly because of its accessibility and lower price but some also find untreated milk having a better taste. Earlier studies have documented that 35–50% of the society in Ethiopia frequently drinks raw milk in its fresh form (39, 40), while a more recent study in central Ethiopia reported 90% of the participants boiling their milk before consumption (41), a difference that could indicate a considerable change in milk consumption habits during the last few years. In addition to raw milk, research has shown that Ethiopians consume a form of fermented milk (locally named *ergo*) at significant rates (41, 42). Under specific conditions, fermented and soured milk has been shown to suppress the growth of *M. bovis* in milk (43, 44), albeit not as successfully as the process of milk pasteurization, which should abolish the risk of zTB in milk. Additionally, a significant proportion of the population in Ethiopia still consumes raw meat (41, 45). Although there were too few zTB cases to draw any strong conclusions on, three out of six participants infected with *M. bovis* in the current study consumed either raw meat or raw milk. Ethiopians’ practice of consuming raw meat and milk can foster favorable conditions for zTB, especially in places where bTB is highly prevalent. However, as discussed above, the high rate of TBLN recorded in Ethiopia cannot be explained by high zTB transmission alone (32).

A significant part of the population in Ethiopia works in the dairy sector. All *M. bovis* cases in the current study were isolated from male study participants. Four out of six *M. bovis* cases had close contact with cattle, likely linked with their occupational status as a veterinarian, an animal attendant, a farmer, or a meat seller. As these occupations are male dominated, this might also explain the absence of female *M. bovis* cases. It has been documented that the risk of zTB increases in areas where bTB is endemic in cattle and where people live under conditions that favor direct contact with infected animals and/or with untreated animal products (7, 46). Therefore, although the risk of contracting zTB is not limited to people working with animals, promoting the use of personal protective equipment by farmers, veterinarians, and abattoir workers may help to reduce the risk of exposure while having contact with *M. bovis*-infected animals. On the other hand, two *M. bovis*-infected cases in this study reported neither an occupational risk nor a consumption habit of untreated milk or raw meat. Although such exposure could still have been possible, these two cases could also have been the result of person-to-person transmission cycles of *M bovis* as previously documented by others (47) or cases of latent infection of this zoonotic pathogen. It is interesting to note that five out of six *M. bovis* infected cases in the current study had PTB and two of them had positive smear results (1+ and 3+), suggesting that *M. bovis* can generate high bacterial loads in sputum, which could have an impact on onwards disease transmission.

A more recent development concerning zTB risks refers not only to *M. bovis* being associated with zTB but that other sub-species of the MTBC may also infect cattle (10) and subsequently transmit to humans. In India, *M. bovis* is rarely found in cattle while *M. orygis* and *M. tuberculosis* are frequently isolated (48, 49). We and others have also isolated *M. tuberculosis* from cattle in Ethiopia (50–52), which is likely to be a spillover from TB in humans (reverse zoonosis). It has been shown experimentally that *M. tuberculosis* is less prone to cause pathology in cattle than *M. bovis* (53, 54), but whether *M. tuberculosis* infected cattle play a significant role in zTB transmission to humans still remains to be answered.

A limitation of this study is the qPCR assays, which are limited in their ability to differentiate between MTBC members other than *M. tuberculosis* and *M. bovis*. Also, the IS*1081* assay is expected to be more sensitive than the RD9 and RD4 assays since it is a multiple copy gene (22). Therefore, samples positive for IS*1081* but negative for RD9 and RD4 by qPCR were likely to have a low bacterial load and consequently a gDNA concentration of MTBC below the limit of detection for the RD9 and RD4 assays. Samples identified only by IS*1081* can then still be either *M. tuberculosis* or *M. bovis*, or even another type of MTBC, affecting the overall prevalence figures presented in this study.

## 5. Conclusion

This Ethiopian study documented a 2.4% prevalence of *M. bovis* in humans, which is higher than those reported in previous large zTB studies in Ethiopia; and we suggest that the higher bTB rates recorded in the dairy sector in central Ethiopia have likely had an impact on the zTB rate in the same area. However, despite sampling in an area with very high bTB prevalence in cattle, the rate of *M. bovis* in this study is still far from the zTB rates reported from Europe at its bTB endemic peak about a century ago. MTBC speciation tools that we used may largely explain this difference. Occupational risk and the widespread habit of raw animal product consumption were noted as possible sources of *M. bovis* infection in humans, while isolation of *M. bovis* from PTB patients also suggests the potential for human-to-human transmission, especially in patients with no known contact history with animals. There is a high chance that patients with *M. bovis* infection get ineffective TB treatment, as *M. bovis* is naturally resistant to pyrazinamide, a first-line drug used for treatment of TB. MTBC species identification should be encouraged, particularly for patients with relapse and treatment failure. New molecular TB diagnostic approaches in the pipeline should be able to differentiate *M. tuberculosis* from *M. bovis* to warrant improved patient management concerning treatment regimens. However, TB control programs need to take into account the additional cost of differential diagnostics in light of the relatively low global burden of zTB. In parallel, stricter adherence to heat-treatment of milk, proper meat inspection, and increased public awareness on the dangers of consuming raw animal products when it comes to zoonotic diseases in general, and zTB in particular, is crucial. Special attention should be given to the occupational risks within the livestock sector, especially in areas where high prevalence of bTB in cattle is well documented.

## Data availability statement

The original contributions presented in the study are included in the article/Supplementary material, further inquiries can be directed to the corresponding authors.

## Ethics statement

The studies involving humans were approved by AHRI/ALERT Ethics Review Committee (AAERC). The studies were conducted in accordance with the local legislation and institutional requirements. The participants provided their written informed consent to participate in this study.

## Members of the ETHICOBOTS consortium

The members of the ETHICOBOTS consortium are: Abraham Aseffa, Adane Mihret, Bamlak Tessema, Bizuneh Belachew, Eshcolewyene Fekadu, Fantanesh Melese, Gizachew Gemechu, Hawult Taye, Rea Tschopp, Shewit Haile, Sosina Ayalew, Tsegaye Hailu, all from the Armauer Hansen Research Institute, Ethiopia; Rea Tschopp from Swiss Tropical and Public Health Institute, Switzerland; Adam Bekele, Chilot Yirga, Mulualem Ambaw, Tadele Mamo, Tesfaye Solomon, all from the Ethiopian Institute of Agricultural Research, Ethiopia; Tilaye Teklewold from Amhara Regional Agricultural Research Institute, Ethiopia; Solomon Gebre, Getachew Gari, Abde Aliy, Abebe Olani, Asegedech Sirak, Gizat Almaw, Getnet Mekonnen, Mekdes Tamiru, Sintayehu Guta, all from the Animal Health Institute, Ethiopia; James Wood (consortium lead author), Andrew Conlan, Alan Clarke, all from Cambridge University, United Kingdom; Henrietta L. Moore and Catherine Hodge, both from University College London, United Kingdom; Constance Smith at University of Manchester, United Kingdom; R. Glyn Hewinson, Stefan Berg, Martin Vordermeier, Javier Nunez-Garcia, all from the Animal and Plant Health Agency, United Kingdom; Gobena Ameni, Berecha Bayissa, Aboma Zewude, Adane Worku, Lemma Terfassa, Mahlet Chanyalew, Temesgen Mohammed, Miserach Zeleke, all from Addis Ababa University, Ethiopia.

## Author contributions

AM, JW, and SB conceived and designed the study. BT, GH, SC, FM, RA, and SA sample collection and laboratory analysis. AM, SA, and SB performed data curation and data interpretation. SA wrote the first draft of the manuscript. AA, AM, JW, RA, SA, and SB reviewed the manuscript. All authors contributed to the article and approved the submitted version.

## Funding

This research was financially supported by the Ethiopia Control of Bovine Tuberculosis Strategies (ETHICOBOTS) project funded by the Biotechnology and Biological Sciences Research Council, the Department for International Development, the Economic & Social Research Council, the Medical Research Council, the Natural Environment Research Council and the Defence Science &Technology Laboratory, under the Zoonoses and Emerging Livestock Systems (ZELS) programme, ref.: BB/S013806/1.

## Conflict of interest

The authors declare that the research was conducted in the absence of any commercial or financial relationships that could be construed as a potential conflict of interest.

## Publisher’s note

All claims expressed in this article are solely those of the authors and do not necessarily represent those of their affiliated organizations, or those of the publisher, the editors and the reviewers. Any product that may be evaluated in this article, or claim that may be made by its manufacturer, is not guaranteed or endorsed by the publisher.

## Supplementary material

SUPPLEMENTARY TABLE S1 qPCR result for culture positive samples.

SUPPLEMENTARY TABLE S2 qPCR results for extracted sputum and FNA (Culture negatives).
